# Distributed Rule-Enabled Interworking Architecture Based on the Transparent Rule Proxy in Heterogeneous IoT Networks

**DOI:** 10.3390/s23041893

**Published:** 2023-02-08

**Authors:** Wenquan Jin, Dohyeun Kim

**Affiliations:** 1 Department of Electronic & Communication Engineering, Engineering College, Yanbian University, Yanji 133002, China; 2 Department of Computer Engineering, Jeju National University, Jeju City 63243, Republic of Korea

**Keywords:** Internet of Things, edge computing, rules engine, transparent computing, proxy, open connectivity foundation, EdgeX

## Abstract

Rule-enabled Internet of Things (IoT) systems operate autonomous and dynamic service scenarios through real-time events and actions based on deployed rules. For handling the increasing events and actions in the IoT networks, the computational ability can be distributed and deployed to the edge of networks. However, operating a consistent rule to provide the same service scenario in heterogeneous IoT networks is difficult because of the difference in the protocols and rule models. In this paper, we propose a transparent rule deployment approach based on the rule translator by integrating the interworking proxy to IoT platforms for operating consistent service scenarios in heterogeneous IoT networks. The rule-enabled IoT architecture is proposed to provide functional blocks in the layers of the client, rule service, IoT service, and device. Additionally, the interworking proxy is used for translating and transferring rules between IoT platforms in different IoT networks. Based on the interactions between the IoT platforms, the same service scenarios are operated in the IoT environment. Moreover, the integrated interworking proxy enables the heterogeneity of IoT frameworks in the IoT platform. Therefore, rules are deployed on IoT platforms transparently, and consistent rules are operated in heterogeneous IoT networks without considering the underlying IoT frameworks.

## 1. Introduction

Internet of Things (IoT) is a paradigm that inspires various industrial domains to deploy a massive number of connected devices to provide intelligent and autonomous services. Based on the IoT platforms, various data are exchanged with the environments through monitoring and controlling the environmental parameters using sensors and actuators [1]. Observing the events in IoT environments enables representing the real world to the cyber world through the data. For reacting to the variety of the IoT data, rules-based approaches can be an efficient mechanism that provides autonomous and dynamic service scenarios to handle the events [2,3,4]. The rule operation in IoT architecture is a computational model that requires functionalities including deployment and managing rules, real-time event processing, and action assignment [5,6,7]. Using the functionalities, the collected sensing data as events to be evaluated based on registered rules and actions are assigned to deliver the command to the actuators for updating the environment.

The rules are a type of context that represents knowledge to operate service scenarios through evaluating with events [8,9,10]. For understanding the knowledge of a rule context, the interpreter must be included in the IoT platform to filter the event based on rule conditions. Therefore, a consistent type of rule context and interface is required to be supported for applying the rules in the IoT platform. In IoT environments, massive data are generated and processed by a number of IoT devices. For an environment, multiple IoT devices are deployed and perform the same functions such as monitoring and controlling, which provides various perspectives of observation and multiple computing resources based on the distributed system of IoT architecture [11]. For reducing the computational complexity, the distributed system assigns tasks to multiple machines to increase the efficiency of the overall system. Additionally, the variety of IoT data is provided through collecting sensing data from the multi-aspect. Therefore, in rule-enabled IoT architecture, the consistent rule context is deployed to the IoT network for operating the same rule scenario in the distributed system using multiple IoT platforms.

The IoT network can be operated by a single rules engine by deploying a rule profile for a rule scenario [12,13,14,15]. Additionally, the rule context can be deployed to each IoT platform in the IoT network for operating the same rule scenario [16,17]. Deploying a consistent rule context to multiple IoT platforms can be performed by a web client to send the rule to each IoT platform and share the rule in the IoT network. However, due to the heterogeneity of the IoT networks, the various IoT frameworks are considered to develop the IoT platforms which have a difficult time operating rules using a consistent type and interface. In heterogeneous IoT networks, multiple protocols are included to support various devices and environments which requires multiple protocol clients for accessing the IoT resources. For operating rules in heterogeneous IoT networks, the rule context must be delivered to each IoT network that is developed in different IoT frameworks. For rule-enabled IoT frameworks, the communication protocol and rule context format can be the issues. The rule context presents the rule knowledge that is used in the rule operator to operate the rule scenario. The knowledge must be interpreted by the rule operator by parsing the context. Moreover, the rule client and server must use the same communication protocol for sending and receiving the rule to deploy in the IoT platform.

However, a variety of IoT frameworks have a difficult time supporting the consistent rule model in diffident IoT networks that are developed using different communication protocols and rule context formats. The interworking proxy can be a solution for translating the protocols between two diffident IoT networks [18,19,20]. As an important network element, the proxy removes the difference in IoT solutions based on the characteristics to enable the consistent data format and service interface [21,22,23]. Through integrating the proxy to the rule-enabled IoT platforms, heterogeneous IoT networks are enabled to provide a consistent rule scenario. Furthermore, rule-enabled IoT platforms are enabled to operate rules transparently in distributed and heterogeneous IoT networks.

For developing distributed rule-enabled interworking in heterogeneous IoT networks, we propose a transparent rule deployment approach based on the rule translator by integrating the interworking proxy to the IoT platforms. The IoT platforms are comprised of IoT and rule services for operating autonomous and dynamic service scenarios. Based on the interactions between the IoT platforms, the computing resources are distributed, and the multi-aspect of sensing data is collected. Additionally, the heterogeneity of IoT frameworks is handled by integrating the interworking proxy to the IoT platforms. Once a rule is deployed in an IoT platform, then the IoT platform delivers to others for synchronizing the rule knowledge to operate the same rule scenario. For synchronizing the rule knowledge in heterogeneous IoT networks, the proposed interworking approach enables the translation of rules between different IoT frameworks and transferring to other IoT networks through destination protocols. Therefore, rules are deployed by the rule client transparently to IoT platforms and operated consistently without considering the underlying IoT frameworks.

In the experiment step, the Open Connectivity Foundation (OCF) and EdgeX frameworks are used for developing two different IoT networks to perform distributed rule-enabled interworking based on providing IoT and rule services in IoT platforms. The frameworks involve different communication protocols and rule models that are handled by the proposed interworking architecture to provide consistent rule operation.

The rest of the paper is structured as follows. Section 2 introduces the related works including existing solutions of rule-enabled IoT frameworks and interworking approaches. Section 3 presents the proposed distributed rule-enabled interworking architecture for heterogeneous IoT networks. Section 4 presents the details of translating and transferring mechanisms based on functional architecture and algorithms. Section 5 introduces the experimental scenarios for the proposed rule interworking mechanism based on OCF and EdgeX. Section 6 presents experimental results and performance evaluation. Finally, we conclude this paper and introduce our future directions in Section 7.

## 2. Related Works

Massive IoT generates great data, such as sensing data, operating mode information, and the actuator status, that trigger further actions to affect the environment in the rule-enabled IoT networks. Many rule-based systems are proposed for providing autonomous and dynamic service scenarios in IoT networks. Mainetti et al. [24] proposed a semantic rule approach to performing events, conditions, and actions for managing IoT devices automatically in the building environment. Lan et al. [25] proposed a universal and suitable rule-based event processing mechanism using the Drools framework for supporting heterogeneous sensing devices. Kulshrestha et al. [26] proposed a real-time monitoring and controlling mechanism based on deploying the rule-based event processor to the network edge. Paganelli et al. [27] proposed the Representational State Transfer (REST)-ful rule management framework that enables multiple levels of configurability and extensibility for providing IoT services.

The EdgeX framework is an edge computing solution for providing various functions through microservices [28]. In the EdgeX framework, the rules engine is provided based on the Kuiper rules engine, which is a lightweight open-source framework using Structured Query Language (SQL)-based rules. The OCF framework is an IoT development specification that is implemented by IoTivity based on the Constrained Application Protocol (CoAP) for constrained IoT devices [29]. The OCF devices include OCF resources to provide services. In the OCF optional specification, the OCF rule server is proposed to provide the autonomous decision logic according to a condition–action pattern. For operating consistent rules in different IoT frameworks, the interworking proxy is important in bridging the IoT platforms. The experiment of the proposed rule interworking approach is performed by using EdgeX and OCF to develop the transparent rule deployment and operation.

For handling the growing data in the rule-based IoT frameworks, the distributed rule-enabled IoT environment overcomes the limitations of resources based on big data processing solutions. Chen et al. [12] proposed a rule engine based on Apache Spark that is a big data processing solution for handling larger event streams in the IoT environment. Wang et al. [30] proposed a distributed rules engine using multiple devices for handling a large amount of data based on the message-passing model for the interoperability in the devices. Mert et al. [31] proposed a visual programming model for defining rules in a distributed IoT environment. Choochotkaew et al. [32] proposed an event-processing mechanism in rule-enabled edge computing based on a pseudo-source mechanism and relation-supportive event specification language for filtering the event stream from multiple edge nodes. However, operating consistent rule scenarios is a challenge for deploying heterogeneous IoT networks in order to implement the distributed rule-enabled IoT environment.

Heterogeneity is not only the challenge of developing software using complex libraries and frameworks in various platforms but also in enabling communications between heterogeneous protocols in IoT networks. For accessing heterogeneous IoT devices without considering underlying protocols, transparent computing enables user-friendly service accesses based on consistent interfaces [33]. Transparent computing enables on-demand cross-platform service access, which supports transparent access to heterogeneous communication solutions [34]. Yoon et al. [35] proposed a proxy for supporting distributed and heterogeneous sensor networks based on deploying the proxy between networks as a middleware to translate request and response messages. Angelo et al. [36] proposed an IoT gateway for mapping a general network protocol to a lightweight protocol for accessing the constrained IoT network transparently. Jeong et al. [37] proposed an IoT architecture providing a transparent discovery to clients without considering while devices move into different networks.

To enable the interworking between the Hypertext Transfer Protocol (HTTP) and OCF networks, the interworking proxy includes the implementation of both protocols, including the HTTP client and server, or the OCF client and server, to request and respond to the messages [38,39]. In the OCF bridge specification, the architecture of the OCF platform is proposed, which forwards the message to different network environments based on the destination protocol client, server, and translator [40]. These studies illustrate that the proxy or gateway enables accessing the heterogeneous network elements transparently.

Table 1 presents the comparisons between the proposed approach and the above-discussed existing approaches.

## 3. Distributed Rule-Enabled Interworking Architecture for Heterogenous IoT Networks

**Figure 1:**
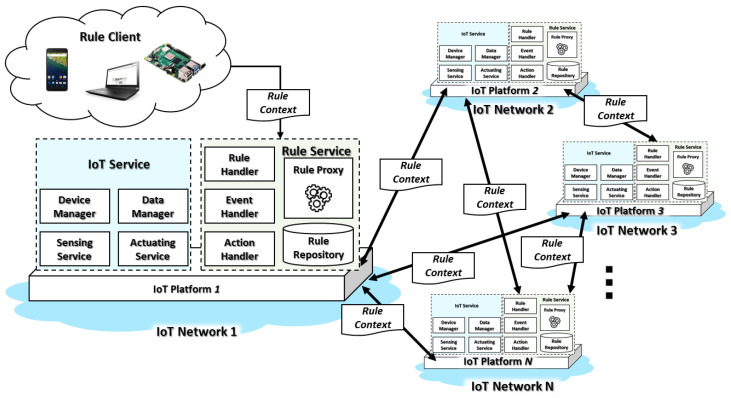
Distributed rule-enabled IoT environment based on heterogeneous IoT platforms.

The proposed IoT architecture comprises multiple heterogeneous IoT networks to provide distributed computing. The rules provide the service scenarios in the IoT environment automatically and dynamically. For synchronizing a consistent rule in the distributed IoT environment, each IoT platform includes IoT and rule services through the functional blocks, as shown in Figure 1.

The distributed rule-enabled IoT architecture includes multiple IoT networks that comprise more than one IoT platform. The IoT networks are deployed using heterogeneous IoT frameworks that provide specific IoT solutions for the IoT environment. For providing interoperability between the IoT networks, the proxies are required to forward the data from an IoT network to another IoT network. In the rule-enabled IoT architecture, the proxy is used for translating and transferring the rule context from an IoT platform to other IoT platforms. The IoT platform includes IoT and rule services that are used for registering IoT devices, collecting data, controlling actuators, deploying service scenarios using rules, and operating the scenarios based on rules. In the IoT service, the device manager, data manager, sensing service, and actuating service can be included to provide the general solution for the IoT environment.

The device manager provides services to register, update, retrieve, and delete the information of IoT devices that represents the actuator sensor and actuator resources for the Internet. The web clients access the represented cyber resources to obtain and input the data to the actual IoT devices. The data manager is used for collecting data from sensing services and sending control commands to the actuating service. The data manager receives the sensing data as events from the sensing service to trigger the rule scenario. Then, the generated action commands are delivered to the actuators through the actuating service for updating the environment.

The rule service includes the rule handler, event handler, action handler, rule proxy, and rule repository to operate the rules for automatically and dynamically deploying and enabling various service scenarios in the IoT environment. The rule handler is used for managing and evaluating rules. The rule clients deploy the rule context through the rule handler that saves the rules to the rule repository. Additionally, updating, deleting, and retrieving services are provided by the rule handler. The event handler observes the sensing data and evaluates the event based on the rule. Once the event satisfies the rule, then the rule handler activates action based on the defined rule context through the action handler. The action handler delivers the action command to the actuator to update the environment. For enabling interworking in heterogeneous IoT networks, the rule proxy includes functions for translating and transferring the rule context to other IoT networks. The rule context is deployed by the rule client, which can be any device with the protocol client for the IoT platform that sends the data to the rule handler through the protocol. Therefore, the rule client is included in IoT platforms for synchronizing the rule context in the IoT network.

**Figure 2:**
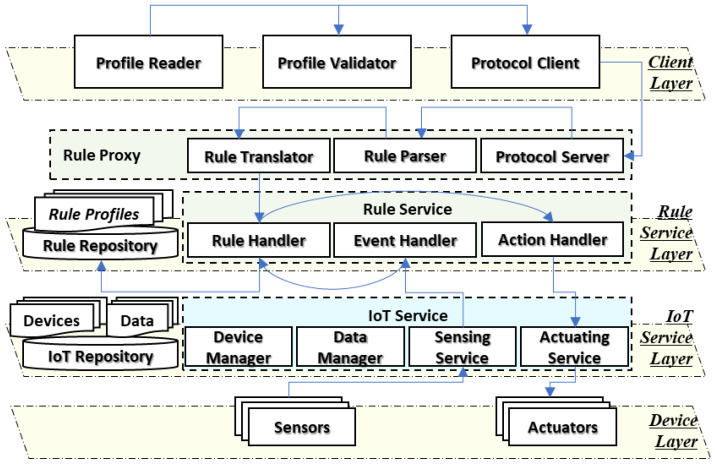
Rule-enabled IoT platform architecture for distributed heterogeneous IoT networks.

For the distributed heterogeneous IoT networks, the proposed rule-enabled IoT platform architecture is presented in Figure 2. The IoT platform architecture comprises the layers of the client, rule service, IoT service, and device. Each IoT platform in the distributed rule-enabled IoT environment includes the specific IoT solution and rule mechanism for providing services in the domain. Nevertheless, the functions can be presented by common titles to depict the details.

The client layer provides functions for reading and validating the rule profile and sending the profile to the destination IoT platform. The profile reader is used for reading the rule context and delivering it to the profile validator. The validator validates the rule context for confirming the availability of the required parameters for operating the rule in the IoT platform. Then, the protocol client sends the rule context to the protocol server in the rule service layer.

The rule service layer provides functions for translating rules for the destination IoT platform and operating rules with events and actions. For the heterogeneous IoT platforms, the rule proxy translates the rule context of other IoT platforms. Once the protocol server receives the rule context through the specific protocol, the rule parser parses the rule context and collects the general information for the current IoT platform. Then, the rule translator translates the rule context for the current IoT platform and delivers the rule to the rule handler, which is a common function for managing the rule context in the IoT platform. The rule repository saves the rule context for evaluating the events and activating actions. Once the event triggers the rule by a delivered event in the event handler, the rule handler sends the command through the action handler that requests the actuating service in the IoT service layer.

The IoT service layer provides functions for managing devices and data and representing sensors and actuators. The IoT devices are registered to the system that represents the actual resource to the Internet based on cyber information. The device and data manager manage the information. The sensing data are collected by sensors and provided by the sensing service. The sensing data are the event that is delivered to the event handler for the rule operation. Once the event is received by the event handler, the rule handler evaluates the event based on the rule context and the results in the control command and object to control the actuators through the actuating services that deliver the control command to the actual actuators.

The device layer provides functions for sensing data and updating the environment. The IoT devices include sensors and actuators for providing IoT services. The interfaces for connecting the actual sensor and actuator units can be wired or wireless. The functions of IoT devices can be part of the IoT platform. Additionally, the functions can be provided through services that bridge between IoT resources and the IoT platform.

## 4. Proposed Transparent Rule Operation Mechanism in IoT Networks

**Figure 3:**
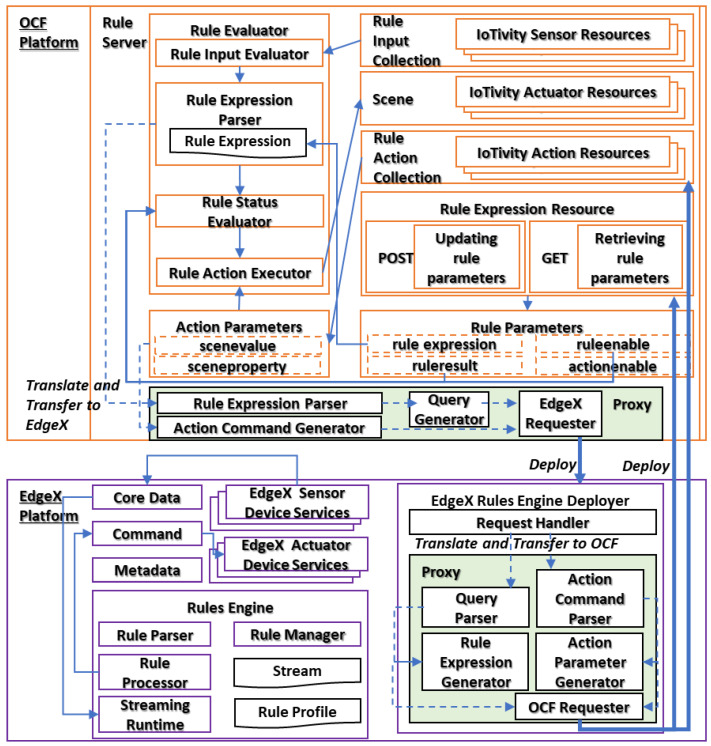
Proposed transparent rule operation functional architecture for IoT platforms.

The OCF platform includes the rule server, which is an OCF server providing services through the OCF communication protocol. The services are provided through the OCF resources for the functions including the rule evaluator, rule input collection, rule action collection, and scene. The EdgeX platform includes core services for managing devices and data. Additionally, the rules engine is included in the EdgeX platform to provide rule operation services. In EdgeX, the services are provided through microservices that expose the functions through REST Application Programming Interfaces (API). Therefore, the functions are intact with each other through the microservices. In the OCF and EdgeX platforms, the functions for translating and transferring to another platform are included, respectively, for the transparent rule operation. Figure 3 shows the proposed transparent rule operation function architecture for the OCF and EdgeX platforms.

In the OCF rule server, the rule evaluator is a function that processes rules with the inputs and conditions. The rule inputs are events that are delivered from the rule input collection. The events are provided to the rule evaluator and evaluated based on the rule expression. The rule status evaluator results in the final decisions for the rule input and delivers the result to the rule action executor that activates the actuators. For receiving the rule inputs, the rule input collection is a set of links that indicate the resources. The resources are sensing resources providing services for receiving events such as sensing data. Through the rule input collection, the events are delivered to the rule evaluator. A scene is a resource that operates the actuator resources.

Once the rule action executor sends the command to the scene, then the actuator resources operate the functions to activate actuator actuators that are represented by the actuator resources. The rule action collection is used for presenting the actions that are concreted by the scene. The rule profile of the OCF platform comprises rule expression and action parameters including the scenevalue and sceneproperty parameters. The rule expression resource provides the interface for receiving the rule expression that is used by the rule expression parser. The action parameters are deployed through the rule action collection and used by the rule action executor. The rule result, ruleenable, and actionenable parameters are used for activating the functions of the OCF rule server. Therefore, the rule expression, scenevalue, and sceneproperty parameters are the translated result of the rule profiles that are operated by other platforms.

The EdgeX core services are provided through the modules of the core data, command, and metadata, which are microservice providers providing services for managing the device and data. The services are invoked by the rules engine to operate the rule scenarios. The sensor device services deliver the sensing data to the core data that send the sensing data as the event to the rules engine to trigger the rule operation. The actuator device service receives the command from the command module for controlling the actuator. The command is generated from the rules engine and sent to the module. The rules engine generates the command based on the rule scenario while the condition is satisfied. The rules engine includes the rule parser, rule manager, rule processor, and streaming runtime functions to operate the rule scenarios.

In the EdgeX rules engine, the stream is a profile that defines the data types of events. The rule profile is used for defining the rules including operating conditions and actions. The streaming runtime catches the events and invokes the rule parser to evaluate the events based on the registered rule profile. The evaluated result is true, and then the rule processor activates the action which is defined in the rule profile. The rule manager is used for managing rule profiles such as deploying, retrieving, and deleting rule profiles. The ExgeX rules engine deployer is used for interacting with the rule manager. The deployer exposes the REST APIs to the Internet for bridging the functions of the rule manager with web clients.

For synchronizing the context of rules in both IoT platforms, the functions of translating and transferring are required to convert and send the rule context from a platform to another platform. In the OCF platform, the translating and transferring function is included in the rule server and receives the rule expression and action parameters. In the EdgeX platform, the translating and transferring function is included in the deployer and receives the rule profile from the request handler.

**Figure 4:**
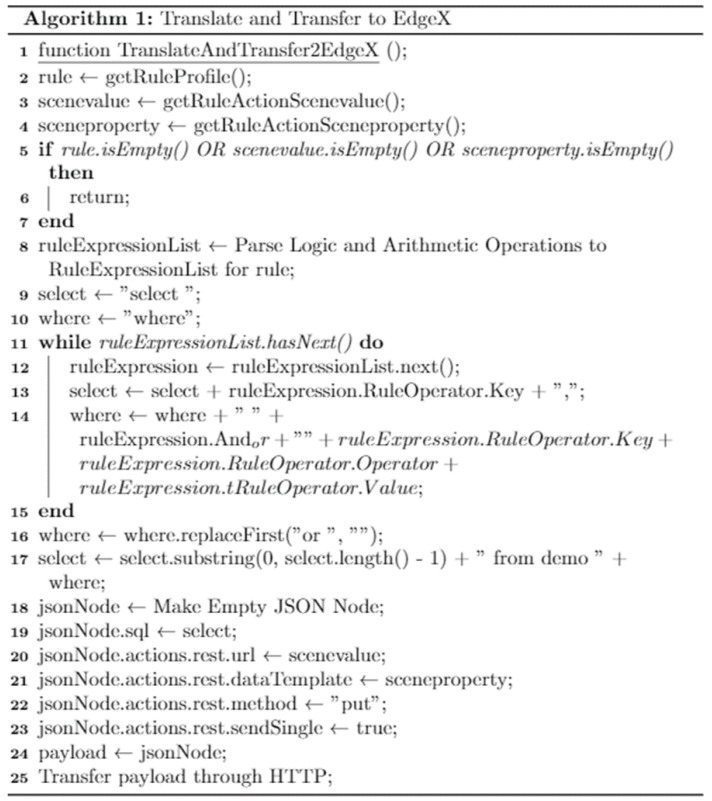
**Algorithm 1** for translating the OCF rule and transferring it to the EdgeX platform.

Figure 4 presents the algorithm of the translating and transferring function in the OCF rule context, which comprises the parameters of rule, scenevalue, and sceneproperty, which are translated to the EdgeX rule. First, the validation of the parameters is performed for parsing the required rule context. The EdgeX rule defines the rule conditions using “select” and “where” based on the SQL format. The rule conditions are combined with the where statement by the loop block. The logical operations “or” and “and” are included in the statement in this step. Finally, the rule context is presented through the JavaScript Object Notation (JSON) node and included in the payload of the request message. The rule context is sent to the EdgeX platform through HTTP.

**Figure 5:**
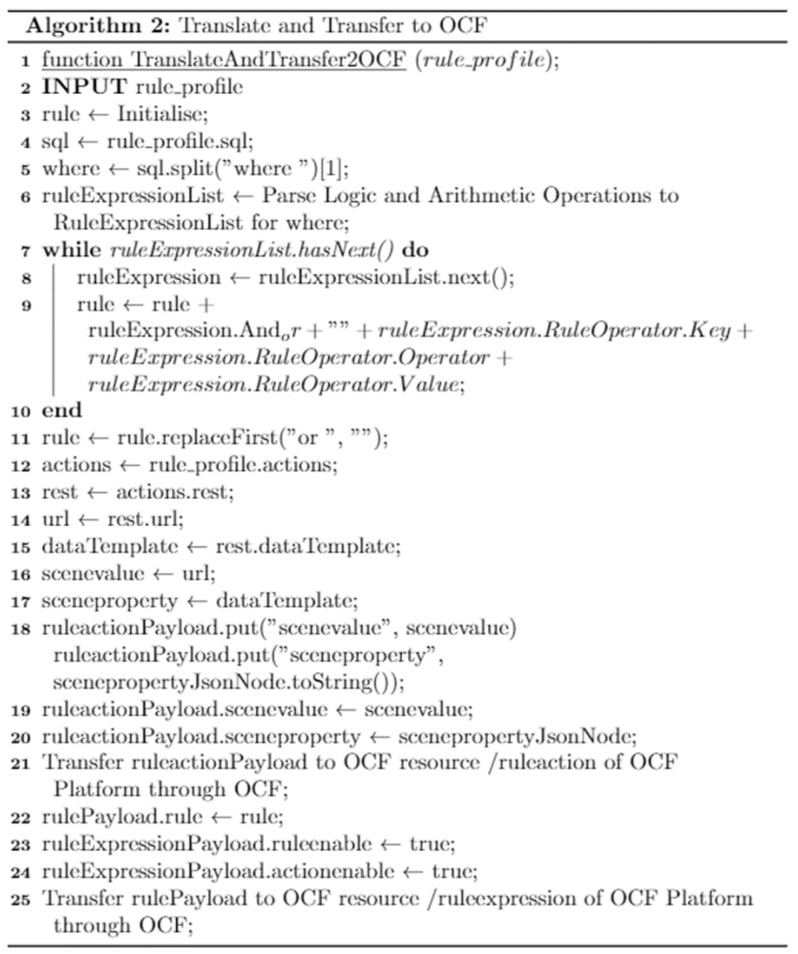
**Algorithm 2** for translating the EdgeX rule and transferring it to the OCF platform.

Figure 5 presents the algorithm of the translating and transferring function in the EdgeX platform that synchronizes the EdgeX rule to the OCF platform. The function is invoked by the EdgeX rules engine deployer from the EdgeX platform once an EdgeX rule is received. The deployer deploys the rule on the EdgeX platform and delivers it to the OCF network based on translating and transferring using the function. The input parameter is rule_profile, which is passed by the deployer service. The parameter value is a rule context in JSON data. For an OCF rule context, the rule, scenevalue, and sceneproperty are required. The rule can be comprised by extracting values from the SQL statement of the EdgeX rule. Using the loop block, conditions are collected and form rule data. In this step, the logical operations are included in the rule. Through parsing the actions property of the EdgeX rule, the scenevalue and sceneproperty are assigned. According to the OCF specification, the OCF resource /ruleexpression handles the rule parameter, and /ruleaction handles the scenevalue and sceneproperty parameters. Additionally, with the rule value, the values of the ruleenable and actionenable parameters are delivered to the /ruleexpression to activate the rule operation.

## 5. Distributed Rule-Enabled Interworking Scenario for the OCF and EdgeX Networks

The distributed rule-enabled interworking scenario is performed based on the OCF and EdgeX networks using the OCF device platform and EdgeX gateway platform, which are the IoT platforms using different IoT protocols and rule frameworks. Based on the proposed scenario, the experimental result is collected and evaluated.

**Figure 6:**
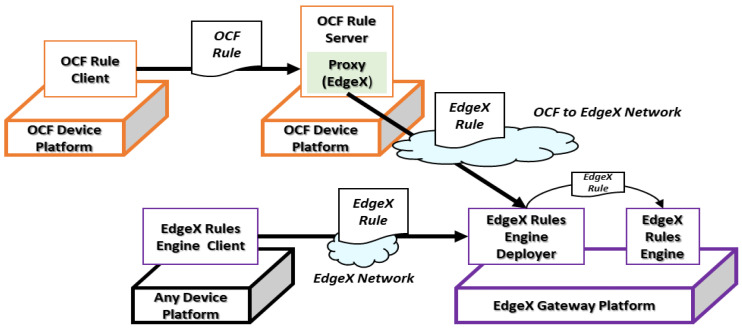
EdgeX gateway platform rule deployment scenario.

Figure 6 shows the deployment scenario for the EdgeX rule that is deployed directly to the EdgeX gateway platform using the EdgeX rules engine client and delivered by the OCF device platform through the proxy to the EdgeX rules engine deployer. The EdgeX rules can be deployed by the EdgeX rules engine client directly. The proxy is used for translating OCF rules to the EdgeX rules and transferring them to the EdgeX gateway platform through HTTP. For deploying the Edge rules to the EdgeX gateway platform, the EdgeX rules engine client sends the rule profile to the deployer from a device platform that can be a platform for running the web client such as web browsers. Then, using the deployer, the EdgeX rule is deployed to the EdgeX rules engine to operate the rule. Once the rule is delivered from the OCF network, the same process is performed. From the OCF network, the EdgeX rule is transferred based on the translation from the OCF rule. In the OCF device platform, the OCF rule is deployed by the OCF rule client. Then, the proxy translates the OCF rule to the EdgeX rule and sends the rule to the EdgeX network.

**Figure 7:**
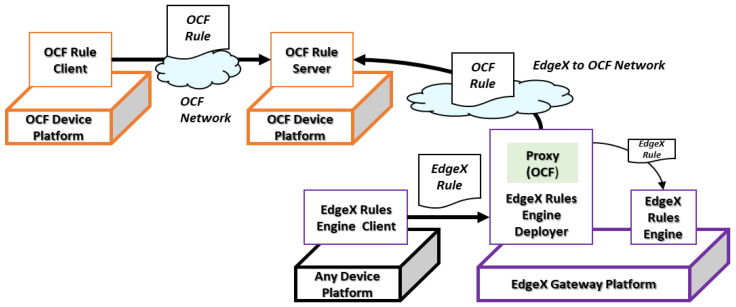
OCF device platform rule deployment scenario.

Figure 7 shows the rule deployment scenario for the OCF device platform. The scenario is performed by deploying the OCF rule to the OCF device platform from the OCF rule client in another OCF device platform and the Edge gateway platform using the EdgeX rules engine deployer. The OCF rule from the OCF rule client is delivered to the OCF rule server through the OCF protocol directly. However, the EdgeX rule cannot be delivered to the OCF rule server through the EdgeX network directly. Therefore, the deployer in the EdgeX gateway platform includes the proxy to translate and transfer the rules between the OCF and EdgeX networks. In this process, the EdgeX rules engine client sends the EdgeX rule to the deployer, which deploys the rule to the EdgeX gateway platform and forwards it to the OCF network.

## 6. Experimental Results and Performance Evaluation

For experimenting with the proposed distributed rule-enabled interworking architecture in heterogeneous IoT networks, the OCF device platform and EdgeX gateway platform are developed to provide IoT and rule services. As presented in Table 2, the experiment is performed by interactions between the entities of the OCF rule server, OCF rule client, EdgeX rules engine deployer, EdgeX rules engine, EdgeX core services, and EdgeX rules engine client. The OCF device platform and EdgeX gateway platform are operated on Ubuntu 20.10 aarch 64 based on Raspberry Pi 4 Model B. The OCF rule server and rule client are operated on the OCF device platform. The IoTivit-lite 2.2.2 framework is used for developing OCF-based sensing, actuating, rule resources in the OCF rule server, and client functions in the OCF rule client. In the EdgeX rules engine deployer, the OCF client and server are included through the IoTitivity framework. For EdgeX services, the EdgeX Hanoi is used for providing IoT and rule services. The version of EdgeX includes the EMQ X Kuiper for providing the rule operation solution. Jetty is a framework for developing the HTTP server in the OCF rule server and deployer. The library httpclient-4.5.13 is used for developing the HTTP client. The Talend API Tester is an online HTTP client that is used for testing the EdgeX-based platform.

**Figure 8:**
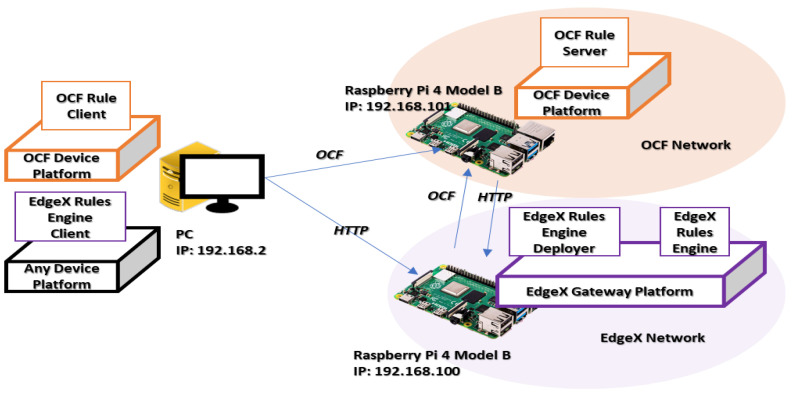
Network environment.

For developing the transparent rule deployment approach, the experimental network environment is configured, as shown in Figure 8, where the proposed network entities are included to perform the proposed distributed rule-enabled interworking. In the experiment, 1 Personal Computer (PC) and 2 Raspberry Pi 4 Model B are deployed. Each hardware machine is assigned an IP and communicates through the router. The OCF rule client is deployed in the PC and sends the rule context through the OCF protocol over the CoAP to the OCF rule server that is deployed in a Raspberry Pi. The EdgeX rules engine client is deployed in the PC and sends the rule context through the HTTP to the EdgeX rules engine deployer that is deployed in another Raspberry Pi. Between two Raspberry Pis, data are also delivered based on the rule translator through the OCF and HTTP.

Table 3 presents the rule context of the OCF platform that is used for operating the rule scenario in the experiment. For the OCF platform, the rule context comprises the parameters rule, sceneproperty, and scenevalue, which are used for defining the rule conditions and actions. The rule context depicts that the event tmp is bigger than 25, and then the status level is updated to be 1 on the IoT resource fan. The resource fan can also be an internal function if the address on the Internet is not defined. For capturing the implementation result of transferring the OCF rule context, the Wireshark network capturing tool is used.

**Figure 9:**
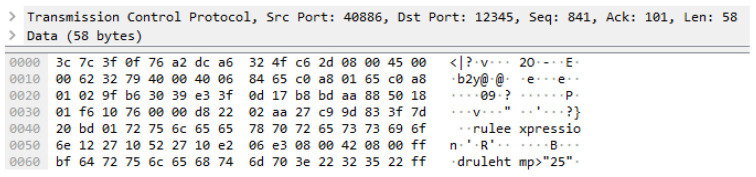
Network packet of rule deployment to the OCF rule server.

The parameter rule of the OCF rule context is captured in the Wireshark, as shown in Figure 9. The message is delivered to the OCF rule server through the OCF protocol that is based on CoAP over the Transmission Control Protocol (TCP). The TCP size is 58 bytes, and the total packet size is 112 bytes.

**Figure 10:**
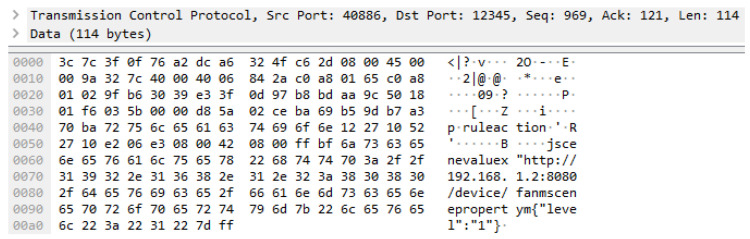
Network packet of sceneproperty and scenevalue deployment to the OCF rule server.

The parameters sceneproperty and scenevalue are delivered to the same resource. Therefore, the parameters and values are captured together, as shown in Figure 10. The TCP size is 114 bytes, and the total packet size is 168 bytes.

**Figure 11:**
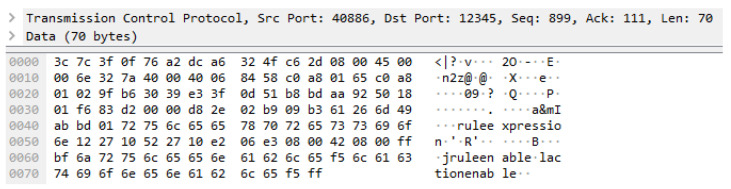
Network packet of enabling a rule on the OCF rule server.

Figure 11 shows the captured network packet of the rule-enabling request that is used for enabling a rule in the OCF rule server. The TCP size is 70 bytes, and the total packet size 124 bytes.

**Figure 12:**
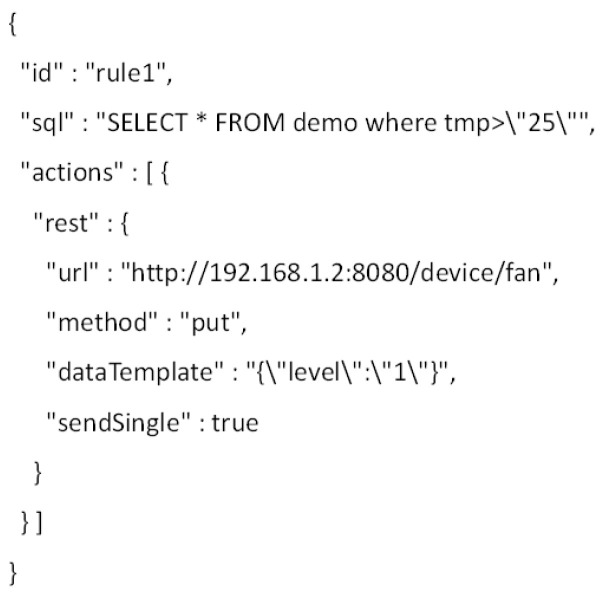
Rule profile for the EdgeX rules engine.

Figure 12 presents the rule context of the EdgeX platform that is used for operating the rule scenario in the experiment. For the EdgeX platform, the rule context is included in a JSON file that has the attributes id, sql, and actions that are assigned values for operating a rule scenario in the EdgeX platform. In the EdgeX platform, multiple rules can be deployed. The attribute id is used for identifying a rule profile. The attribute sql is used for defining the conditions for events. The actions attribute is used for defining the activated actuator details and commands. The rule profile includes the same context as the OCF rule context.

**Figure 13:**
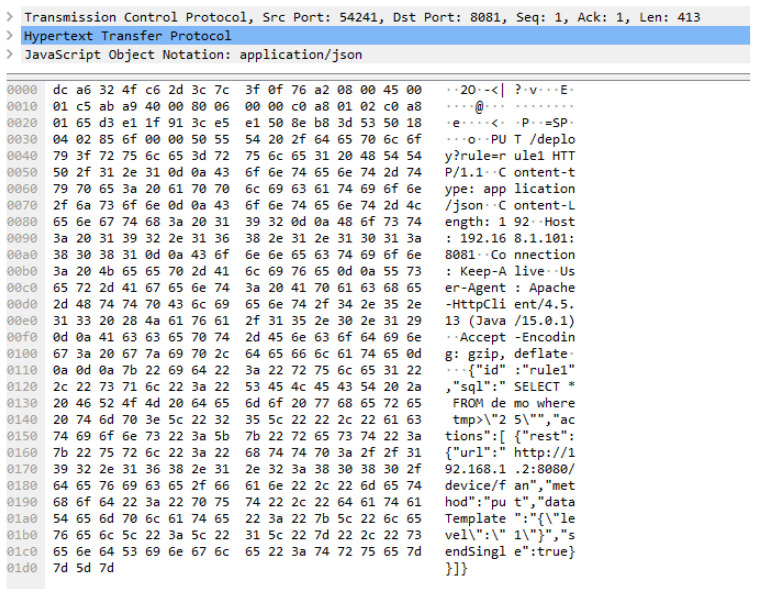
Network packet of deployment to the EdgeX rule engine.

Figure 13 shows the captured network packet of the rule profile for the EdgeX platform. The message is delivered through HTTP to the EdgeX rules engine deployer from the client. The payload includes JSON data that are constructed for the EdgeX rule profile in the experiment. The size of the TCP is 413 bytes, and the total packet size is 467 bytes.

For evaluating the performance of the proposed distributed rule-enabled IoT environment, the network packet size and Round-Trip Time (RTT) are collected for the OCF and EdgeX platforms. The collected results can be referred to in further studies.

**Figure 14:**
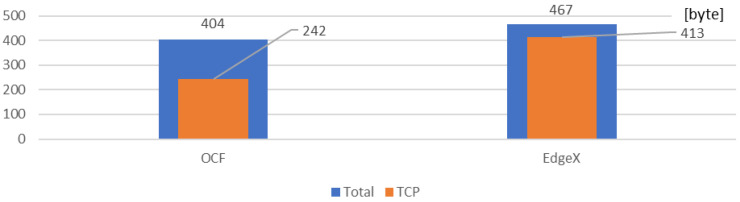
Network packet size comparison for the OCF rule server and EdgeX rules engine.

Figure 14 shows the packet size of the OCF and EdgeX rule context. The deployed rules in the OCF and EdgeX platforms are used for the same rule scenario. As depicted in the implementation results, the OCF rule is delivered in three packets, and the EdgeX rule is delivered by one packet. The total size of the OCF rule is 404 bytes, which are delivered through the OCF protocol. The total size of the EdgeX rule is 467 bytes, which are delivered through HTTP. The size of the packets can be referred to in further developments. For example, in the constrained environment, the OCF network can be deployed because the network cost is less than that of the EdgeX platform.

**Figure 15:**
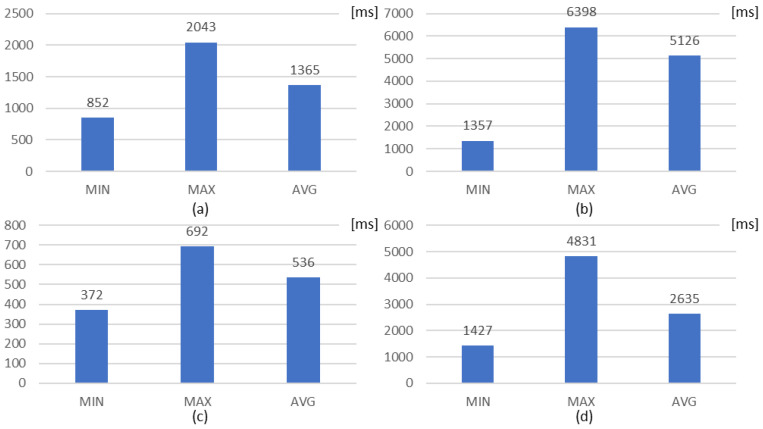
Rule deployment delays: (**a**) OCF rule client to OCF rule server; (**b**) EdgeX rules engine client to OCF rule server; (**c**) EdgeX rules engine client to EdgeX rules engine; (**d**) OCF rule client to EdgeX rule engine.

Figure 15 shows the rule deployment delays, including the RTT for sending the rule from the OCF rule client to the OCF rule server, from the EdgeX rules engine client to the OCF rule server, from the EdgeX rules engine client to the EdgeX rules engine, and from the OCF rule client to the EdgeX rule engine. Figure 15a,c are collected for direct request without the interworking proxy. However, the OCF rule server takes time to process the request. Figure 15b,d are collected over the OCF and EdgeX networks, which takes more time.

In the proposed distributed rule-enabled interworking architecture, the latency of translating and transferring takes time due to the disadvantage of the OCF framework. Therefore, the performance of the rule translator is difficult to achieve in a real-time process. Nevertheless, the evaluation results can be considered in further developments for transparent rule-enablement in heterogeneous IoT environments. Additionally, the network packets can be considered to make the strategy for deploying the IoT networks to constrained and non-constrained environments.

## 7. Conclusions and Future Directions

In this paper, we proposed a distributed rule-enabled interworking architecture based on the rule interworking proxy in the IoT platforms to provide transparent rule deployment and operation in heterogeneous IoT networks. For developing the proposed rule-enabled IoT architecture, the OCF and EdgeX frameworks are used for providing IoT and rule services that deliver the data through CoAP and HTTP because the frameworks involve different communication protocols and rule models. The IoT platforms comprise IoT and rule services for operating autonomous and dynamic service scenarios based on real-time events and actions in heterogeneous IoT networks. However, the interactions are performed between the IoT platforms based on different IoT frameworks. The integrated rule interworking proxy enables rules to be operated for the same service scenarios in the IoT environment. Therefore, the rules are deployed transparently for operating consistent rule scenarios in heterogeneous IoT networks without considering the underlying IoT frameworks. According to the real environment experiment, the performance of the experiment presents that the latency of translating and transferring takes time. The network packets can be considered to make the strategy for deploying the IoT networks to constrained and non-constrained environments.

In future directions, we will separate the interworking proxy from the IoT platform for serving multiple IoT frameworks in a standalone entity. Additionally, a general rule context will be used in the translation process. Therefore, the interworking proxy manages the rules and translation processes in heterogeneous IoT networks in order to operate consistent rule scenarios. Moreover, framework-specific properties of rules can be considered in the translation process for transferring more completed rules to the destination IoT networks.

## Figures and Tables

**Table 1:** Comparison with existing approaches.

Title	Method	Application
Mainetti et al. [24]	Semantic Rule Approach	Buildings
Lan et al. [25]	Drools-Based Universal Rules Engine	Sensor Network
Kulshrestha et al. [26]	Real-Time Rule-Processing Mechanism	Edge Computing
Chen et al. [12]	Large Event Stream Handling	IoT Environment
Choochotkaew et al. [33]	Pseudo-Source-Based Rule Modeling	Edge Computing
Proposed Approach	Transparent Rule Proxy	Heterogeneous IoT Networks

**Table 2:** Experimental environment.

Platform	Entity	Hardware	OS	Library and Framework
OCF Device Platform	OCF Rule Server	Raspberry Pi 4 Model B	Ubuntu 20.10 aarch 64	IoTivit-lite 2.2.2, Jackson 2.11.4, jetty-9.4.40v20210413, httpclient-4.5.13, javax.servlet-api-2.11.4
	OCF Rule Client			IoTivit-lite 2.2.2, Jackson 2.11.4
EdgeX Gateway Platform	EdgeX Rules Engine Deployer			IoTivit-lite 2.2.2, Jackson 2.11.4, jetty-9.4.40v20210413, httpclient-4.5.13, javax.servlet-api-2.11.4
	EdgeX Rules Engine			EMQ X Kuiper for EdgeX Framework Hanoi
	EdgeX Core Services			EdgeX Framework Hanoi
Web Client Platform	EdgeX Rules Engine Client	PC (i9-10900)	Windows 10 64 bit	Talend API Tester

**Table 3:** Rule profile for the OCF rule server.

Parameter	Value
rule	tmp > “25”
sceneproperty	{“level”:”1”}
scenevalue	http://192.168.1.2:8080/device/fan
Parameter	Value

## Data Availability

Data sharing not applicable.
